# Immunosuppressives discontinuation after renal response in lupus nephritis: predictors of flares, time to withdrawal and long-term outcomes

**DOI:** 10.1093/rheumatology/keae381

**Published:** 2024-07-22

**Authors:** Alexandros Panagiotopoulos, Eleni Kapsia, Ioannis El Michelakis, John Boletis, Smaragdi Marinaki, Petros P Sfikakis, Maria G Tektonidou

**Affiliations:** Rheumatology Unit, First Department of Propaedeutic Internal Medicine, Joint Academic Rheumatology Program, Laiko Hospital, Medical School, National & Kapodistrian University of Athens, Athens, Greece; Department of Nephrology and Renal Transplantation, Laiko Hospital, Medical School, National and Kapodistrian University of Athens, Athens, Greece; Department of Nephrology and Renal Transplantation, Laiko Hospital, Medical School, National and Kapodistrian University of Athens, Athens, Greece; Department of Nephrology and Renal Transplantation, Laiko Hospital, Medical School, National and Kapodistrian University of Athens, Athens, Greece; Department of Nephrology and Renal Transplantation, Laiko Hospital, Medical School, National and Kapodistrian University of Athens, Athens, Greece; Rheumatology Unit, First Department of Propaedeutic Internal Medicine, Joint Academic Rheumatology Program, Laiko Hospital, Medical School, National & Kapodistrian University of Athens, Athens, Greece; Rheumatology Unit, First Department of Propaedeutic Internal Medicine, Joint Academic Rheumatology Program, Laiko Hospital, Medical School, National & Kapodistrian University of Athens, Athens, Greece

**Keywords:** lupus nephritis, immunosuppressive agents, immunosuppressive tapering, discontinuation, flares, long-term outcomes, renal failure

## Abstract

**Objectives:**

The optimal duration of immunosuppressive (IS) treatment for lupus nephritis (LN) remains uncertain. We assessed the prevalence and predictors of IS tapering and discontinuation (D/C) in LN patients.

**Methods:**

Data from 137 inception cohort LN patients were analysed. We examined determinants of flares during tapering and after IS D/C, D/C achievement and time to D/C, and adverse long-term outcomes applying logistic and linear regression models.

**Results:**

IS tapering was attempted in 111 (81%) patients, and D/C was achieved in 67.5%. Longer time to achieve complete renal response (CR) [odds ratio (OR): 1.07, *P* = 0.046] and higher SLEDAI-2K at tapering initiation (OR: 2.57, *P* = 0.008) were correlated with higher risk of renal flares during tapering. Persistent hydroxychloroquine use (≥2/3 of follow-up) (OR: 0.28, *P* = 0.08) and lower SLEDAI-2K 12 months before IS D/C (OR: 1.70, *P* = 0.013) decreased the risk of post-D/C flares. Adverse outcomes (>30% estimated glomerular filtration rate decline, chronic kidney disease, end-stage renal disease, death) at the end of follow-up (median 124 months) were more frequent in patients with flares during IS tapering (53% *vs* 16%, *P* *<* 0.0038) but did not differ between IS D/C achievers and non-achievers. In proliferative LN, differences mirrored those in the entire cohort, except for time to D/C, which occurred 20 months earlier in membranous *vs* proliferative LN (β = −19.8, *P* = 0.014).

**Conclusion:**

Earlier CR achievement and lower SLEDAI-2K at tapering initiation prevent flares during IS tapering, while persistent hydroxychloroquine use and lower SLEDAI-2K 12 months before IS D/C prevent post-D/C flares. Flares during tapering increase the risk of unfavourable long-term outcomes. Earlier IS D/C is feasible in membranous LN.

Rheumatology key messages

- Earlier complete renal response, lower SLEDAI-2K and persistent HCQ use are crucial for a safe tapering strategy.
- Adverse long-term renal outcomes are mainly associated with renal flares during immunosuppression tapering.
- Earlier discontinuation of immunosuppression can be considered in membranous lupus nephritis.

## Introduction

Lupus nephritis (LN) is a severe manifestation of systemic lupus erythematosus (SLE) affecting 25–60% of patients and jeopardizing renal and overall survival [1–5]. Standard immunosuppression (IS) consists of an initial phase aimed at achieving renal remission, followed by a ‘maintenance’ phase targeting sustained remission and flare prevention while minimizing treatment-related adverse effects. However, the optimal duration of maintenance treatment remains unclear.

Flare rates in the LN literature range from 8% at 1 year to 48% at 10 years, and from 18% to 38.5% after IS discontinuation(D/C) [6–17]. Notably, renal flares have been associated with shorter kidney lifespan, treatment escalation, impaired health-related quality of life and socioeconomic implications [18, 19]. The updated 2023 EULAR and the 2024 KDIGO guidelines emphasize the importance of continuing IS treatment for at least 3 years after renal response, with gradual tapering before withdrawal [20, 21]. Nevertheless, little is known about the timing and predictors of safe initiation of IS tapering, timing of IS D/C, its correlation with the risk of post-IS D/C flares, and adverse renal outcomes.

Herein, we aimed to examine (i) the rates of IS tapering and IS D/C achievement; (ii) risk factors for flares (renal and extrarenal) during tapering and after IS D/C; (iii) predictors of D/C achievement and time to D/C post-renal response; and (iv) the impact of IS tapering and D/C on long-term renal outcomes in patients with LN.

## Methods

### Study population

Data from two inception cohorts of 142 patients with LN diagnosed between 1992 and 2021 at two joint academic centres (Nephrology and Rheumatology Units, Laiko General Hospital of Athens) were examined. Patients with end-stage renal disease (ESRD) at the time of diagnosis (*n* = 1) and those treated only with glucocorticoids (GCs) (*n* = 4, all with membranous LN) were excluded from the study. All patients had biopsy-proven LN (class III, IV, V, III/IV+V) according to the 2003 ISN/RPS LN classification system [22] and met the 2019 SLE classification criteria [23]. The study was approved by the Institutional Review Board (protocol number 14697/08-11-2022) and did not include any identifiable patient data.

### Data collection

The clinical, laboratory, histological and treatment data for each patient were recorded at the time of LN diagnosis (baseline) and at 3, 6, 9, 12, 24, 36, 48, 60 and 72 months thereafter (Supplementary Data S1, available at *Rheumatology* online). Additional data were collected upon initiation of IS tapering, 12 months before D/C, at the time of IS D/C, and at the last follow-up visit. Renal response and flares were defined according to the 2012 EULAR/ERA-EDTA guidelines [24] and the 2012 KDIGO recommendations [25]. SLE activity and remission targets were evaluated using the Lupus Low Disease Activity State (LLDAS) and the DORIS clinical and DORIS complete remission (Supplementary Data S2, available at *Rheumatology* online) [26–30].

Tapering was defined as any reduction in IS dosage [excluding GCs and hydroxychloroquine (HCQ)] following (partial or complete) renal response [24, 25]. IS dose reduction due to drug toxicity or poor compliance was not considered IS tapering. Only patients who underwent IS tapering because of stable disease were evaluated for IS D/C achievement. The time to IS tapering initiation and IS D/C was calculated after the attainment of the first complete renal response (CR); in four patients only partial response was achieved; and thus, this time point was used in these cases.

We also assessed the achievement of disease modification (DM), defined as meeting all four specific criteria [24-h proteinuria, estimated glomerular filtration rate (eGFR), renal flares and GCs] across three time frames: year 1, years 2–5 and beyond 5 years [31, 32].

### Statistical analysis

Continuous variables are expressed as median and interquartile range (IQR) due to their non-normal distribution, and categorical variables are presented as frequencies and percentages. The Mann–Whitney *U*-test was used to compare continuous variables between groups, while the χ^2^ test was used to compare categorical variables when their assumptions were met; otherwise, Fisher's exact test was employed. Logistic regression models were used to investigate the predictors of flares, and linear regression analysis models to determine the factors affecting the time to IS D/C. Kaplan–Meier survival curves were generated to estimate the composite outcome of ESRD or death, as these represent the primary hard outcomes in LN. Odds ratios (ORs), β-coefficients, *P*-values and 95% CIs for the univariate and multivariate models are presented (details in Supplementary Data S3, available at *Rheumatology* online). Data analysis was conducted using Stata 18.0 software (StataCorp., College Station, TX, USA). All tests were two-tailed, and significance was set at α = 0.05.

## Results

In total, 137 patients were included in this study (flow chart given in Supplementary Fig. S1, available at *Rheumatology* online). Baseline clinical/laboratory, treatment and histological characteristics of the patients, and clinical status at 12 months after LN diagnosis are presented in Table 1.

**Table 1.** Demographic, clinical, laboratory, histological characteristics and immunosuppressive regimens at baseline and 12 months after diagnosis

Characteristic	Value
At baseline	
Age, median (IQR), years	32 (19)
Females, *n* (%)	112 (81.7)
Ethnicity (White), *n* (%)	133 (97)
SLE duration before LN^a^, median (IQR), years	5 (8)
LN as first presentation of SLE, *n* (%)	70 (51)
Hypertension, *n* (%)	32 (23.7)
eGFR, median (IQR), ml/min/1.73 m^2^	96 (44.5)
≤30, *n* (%)	12 (8.8)
31–60, *n* (%)	16 (11.8)
>60, *n* (%)	108 (79.4)
Proteinuria, median (IQR), g/24 h	3 (4)
≤1, *n* (%)	29 (21)
1–3, *n* (%)	44 (32)
>3, *n* (%)	63 (47)
Active urine sediment	118 (86.8)
LN class, *n* (%)	
III	28 (20.4)
IV	46 (33.6)
III, IV+V	25 (18.3)
V, II+V	38 (27.7)
SLEDAI-2K, median (IQR)	12 (6)
Low C3, *n* (%)	85 (72.6)
Low C4, *n* (%)	77 (65.8)
Positive anti-ds DNA	93 (83)
Activity Index^b^, median (IQR)	10 (6)
Chronicity Index^b^, median (IQR)	2 (2)
Interstitial fibrosis/Tubular atrophy^c^, *n* (%)	
None/mild	120 (90)
Moderate/severe	13 (10)
Number of crescents, median (IQR)	0 (3)
Glomerulosclerosis^d^, median (IQR), %	7 (18)
Induction treatment, *n* (%)	
Cyclophosphamide^e^	81 (63.8)
Mycophenolic acid^f^	47 (36.2)
Maintenance treatment, *n* (%)	
Mycophenolic acid	110 (80.3)
Azathioprine	16 (11.7)
Other	11 (8)
12 months post diagnosis	
Proteinuria, median (IQR), g/24 h	0.23 (0.6)
≤0.8, *n* (%)	91 (79)
>0.8, *n* (%)	24 (21)
eGFR, median (IQR), ml/min/1.73 m^2^	108.5 (31.5)
SLEDAI-2K, median (IQR)	4 (5)
CR, *n* (%)	94 (69)

a Only for patients with SLE-related manifestations diagnosed prior to LN.

b For proliferative LN.

c Refers to the percentage of the renal cortex involved by interstitial fibrosis and tubular atrophy.

d Percentage of sclerosed glomeruli among the total number of glomeruli.

e In combination with rituximab in 12/81.

f In combination with rituximab in 7/47. anti-dsDNA: antibodies against double-stranded DNA; CR: complete remission; eGFR: estimated glomerular filtration rate using the CKD-EPI formula; IQR: interquartile range; LN: lupus nephritis; SLE: systemic lupus erythematosus; SLEDAI-2K: systemic lupus erythematosus disease activity index.

### Initiation of immunosuppression tapering

Immunosuppression tapering was attempted in 111 (81%) LN patients at a median time of 34 (IQR: 40) months post-renal response, and 43.5 (IQR: 38) months after the initiation of induction treatment. Similar percentages of tapering attempt (79% *vs* 86% *P* = 0.34) and comparable median time post LN diagnosis (43 *vs* 42 months, *P* = 0.85) were observed between patients with proliferative and pure membranous LN. The reasons for not attempting IS tapering (*n* = 26) are described in Supplementary Data S4a, available at *Rheumatology* online.

At IS tapering initiation, the median 24-h proteinuria was 0.17 g and the median eGFR was 110 ml/min/1.73 m^2^. Sixty-one percent of patients were receiving HCQ, and 45.7% had discontinued GCs. Patients still receiving GCs were administered a median dose of 4 mg/day. Almost all patients met the criteria for LLDAS and DORIS clinical remission (96.8% and 95.7%, respectively); however, only half of them were on DORIS complete remission (49.5%).

### Flares during immunosuppression tapering

Among the 111 patients in whom IS tapering was attempted, 19 (17.1%) exhibited flares during tapering. Eleven patients experienced renal flares, and nine had extrarenal flares: CNS involvement in 1/9, myositis in 1/9, arthritis in 6/9 and arthritis with rash in 1/9. One patient had both renal and extrarenal flares (which are described in Supplementary Data S5, available at *Rheumatology* online). Flares were treated following established treatment protocols, leading to re-achievement of remission in all patients. Patients who experienced renal flares during tapering differed in LN class and induction treatment; 10 of 11 patients who relapsed had proliferative LN *vs* 1 with membranous LN (*P* *=* 0.16), and 9 of 11 patients received cyclophosphamide (CYC) at baseline *vs* none treated with mycophenolic acid (MPA) (*P* *=* 0.02, Table 2) (one was treated with rituximab and one with ciclosporin).

**Table 2.** Differences between patients who experienced renal flares during tapering and those who did not

Characteristic	Total (*n* = 111)	Without flare (*n* = 100)	With flare (*n *= 11)	*P*-value	Multivariate analysis, OR (95% CI), *P*-value
At baseline					
Age, median (IQR), years	32 (19)	32.5 (17.5)	30 (21)	0.55	
Females, *n* (%)	89 (80.2)	81 (81)	8 (72.7)	0.45	
eGFR, median (IQR), ml/min/1.73 m^2^	96 (43)	96 (45)	103 (35)	0.94	
Proteinuria, median (IQR), g/24 h	3 (4.2)	2.85 (4.1)	3.5 (3.8)	0.18	
Active urine sediment, *n* (%)	98 (88.3)	87 (87)	11 (100)	0.35	
LN class, *n* (%)	78 (70.3)	68 (68)	10 (90.9)	0.16	
III	25 (22.5)	22 (22)	3 (27.3)	0.31	
IV	37 (33.3)	31 (31)	6 (54.5)		
III, IV+V	16 (14.4)	15 (15)	1 (9.1)		
V, II+V	33 (29.7)	32 (32)	1 (9.1)		
SLEDAI-2K, median (IQR)	12 (5)	12 (6)	12 (4)	0.33	
Low C3, *n* (%)	68 (73.1)	62 (72.9)	6 (75)	0.90	
Low C4, *n* (%)	65 (69.9)	59 (69.4)	6 (75)	0.74	
Positive anti-ds DNA, *n* (%)	78 (85.7)	73 (85.9)	5 (83.3)	0.86	
Activity Index^a^, median (IQR)	10 (6)	10 (5)	8 (7)	0.50	
Chronicity Index^a^, median (IQR)	2 (2)	2 (2)	1 (3)	0.30	
Interstitial fibrosis/tubular atrophy (moderate–severe)^b^, *n* (%)	10 (9.3)	8 (8.2)	2 (18.2)	0.27	
Number of crescents, median (IQR)	3 (3)	3 (3.5)	5 (4)	0.17	
Glomerulosclerosis (%)^c^, median (IQR)	8.7 (18)	8.7 (15)	0 (18)	0.51	
Induction treatment (cyclophosphamide), *n* (%)	66 (66)	57 (57)	9 (82)	**0.02**	
Maintenance treatment (mycophenolic acid), *n* (%)	86 (85)	79 (85)	7 (78)	0.61	
12 months post diagnosis					
Proteinuria, median (IQR), g/24 h	0.26 (0.5)	0.2 (0.38)	0.85 (2.5)	**0.02**	
≤0.8, *n* (%)	77 (79.4)	72 (82.8)	5 (50)	**0.03**	
>0.8, *n* (%)	20 (20.6)	15 (17.2)	5 (50)		
eGFR, median (IQR), ml/min/1.73 m^2^	109 (30)	109 (29)	90 (41)	0.10	
SLEDAI-2K, median (IQR)	4 (5)	2 (4)	4 (5)	**0.03**	
Complete remission, *n* (%)	81 (73.6)	76 (76.7)	5 (45.5)	**0.02**	
Disease modification, *n* (%)	77 (81)	72 (84.7)	5 (45.5)	**0.01**	
At tapering initiation					
eGFR, median (IQR), ml/min/1.73 m^2^	110 (41)	110 (40)	107 (43)	0.82	
Proteinuria, median (IQR), g/24 h	0.17 (0.14)	0.16 (0.14)	0.19 (0.23)	0.55	
HCQ use, *n* (%)	57 (60.6)	54 (63.5)	3 (33.3)	0.07	
GC use, *n* (%)	51 (54.3)	44 (51.2)	7 (87.5)	0.06	
GC, median (IQR), mg/24 h	4 (2)	4 (1)	4 (2)	0.54	
SLEDAI-2K, median (IQR)	0 (2)	0 (2)	2 (2)	**0.046**	**1.28 (5.16, 2.57), 0.008**
LLDAS, *n* (%)	90 (96.8)	83 (97.6)	7 (87.5)	0.24	
DORIS clinical remission, *n* (%)	89 (95.7)	82 (96.5)	7 (87.5)	0.30	
DORIS complete remission, *n* (%)	46 (49.5)	44 (51.8)	2 (25)	0.14	
Time to first CR, median (IQR), months	7.5 (8.5)	7 (8.5)	12.5 (24)	**0.03**	1.01 (1.15, 1.07), 0.046
Sustained CR, median (IQR), months	31 (27)	31 (24)	22 (41)	0.93	
Renal response to tapering initiation, median (IQR), months	34 (40)	33 (36)	52 (22)	0.12	
LN diagnosis to tapering initiation, median (IQR), months	43.5 (38)	43 (37)	50 (49)	0.12	
At least one renal flare before tapering, *n* (%)	23 (20.7)	18 (18)	5 (45.5)	**0.03**	
Extrarenal flare before tapering, *n* (%)	5 (4.8)	5 (5.4)	0 (0)	0.43	

Statistical significance (*P < *0.05) is shown in bold.

a For proliferative LN.

b Refers to the percentage of the renal cortex involved by interstitial fibrosis and tubular atrophy.

c Percentage of sclerosed glomeruli among total number of glomeruli. anti-ds DNA: antibodies against double-stranded DNA; CR: complete remission; DORIS: Definition of Remission In Systemic Lupus Erythematosus; eGFR: estimated glomerular filtration rate using the CKD-EPI formula; GC: glucocorticoid; HCQ: hydroxychloroquine; IQR: interquartile range; LLDAS: Lupus Low Disease Activity State; LN: lupus nephritis; OR: odds ratio; SLEDAI-2K: systemic lupus erythematosus disease activity index.

Patients without renal flares during tapering were more likely to achieve CR (76.7% *vs* 45.5%, *P* *=* 0.02), lower SLE Disease Activity Index 2000 (SLEDAI-2K) (median 2 *vs* 4, *P* *=* 0.03), lower 24-h proteinuria (median 0.2 *vs* 0.85 g, *P* *=* 0.02), and lower than 0.8 g/24-h proteinuria (82.8% *vs* 50%, *P* *=* 0.03) at 12-month follow-up. Interestingly, 84.7% of patients without flares *vs*. 45.5% (*P* *=* 0.01) of those who relapsed during the IS tapering period met the DM criteria at year 1 (Table 2).

Patients who experienced renal flares during tapering (*vs* those who did not) were more likely to have higher SLEDAI-2K scores (median 2 *vs* 0, *P* *=* 0.046), not achieve complete DORIS remission (25% *vs* 51.8%, *P* *=* 0.14), not use HCQ (33.3% *vs* 63.5%, *P* *=* 0.07), and still receive GCs (87.5% *vs* 51.2%, *P* *=* 0.06) at the time of tapering initiation (Table 2).

Patients with at least one renal flare before tapering were more likely to experience another renal flare during tapering (45.5% *vs* 18%, *P = *0.03). Neither the time between IS treatment initiation and tapering initiation nor the time between renal response and tapering initiation differed between patients with and without renal flares during tapering (median 43 *vs* 50 months, *P = *0.12; 33 *vs* 52 months, *P = *0.12, respectively). Although the duration of CR before tapering initiation was comparable, the time to first CR was significantly shorter for patients who did not experience flares during tapering *vs* those who did (median 7 *vs* 12.5 months, *P = *0.03) (Table 2).

Time to first CR and SLEDAI-2K score at tapering initiation were the only variables included in the multivariate analysis because of the limited number of flares during tapering (Supplementary Data S3 and Supplementary Table S1, available at *Rheumatology* online). Notably, both longer time to achieve first CR (OR: 1.07, *P = *0.046) and higher SLEDAI-2K score at tapering initiation (OR: 2.57, *P = *0.008) were independently associated with renal flares during IS tapering.

In proliferative LN, the differences between patients with and without renal flares during tapering were similar to those observed in the total cohort. Notably, the SLEDAI-2K scores were comparable, and pre-tapering renal flares did not affect the risk of renal flares during IS tapering (Supplementary Table S3, available at *Rheumatology* online).

### Immunosuppression discontinuation achievement

Seventy-five (67.5%) of the 111 patients who initiated tapering achieved IS D/C at a median time of 57 months (IQR: 43) post-renal response. Similar percentages of IS D/C were observed between patients with proliferative and pure membranous LN (69% *vs* 64%, *P = *0.65). IS D/C achievers and non-achievers had comparable characteristics at baseline, 12 months after diagnosis, and at the time of initiation of IS tapering. The only difference was the incidence of flares (either renal or extrarenal) during tapering; non-achievers relapsed more frequently during IS tapering in both the entire cohort (proliferative and pure membranous LN) (27.8% *vs* 12%, *P = *0.039) and the proliferative LN group separately (37.5% *vs* 11%, *P = *0.006). Both achievers and non-achievers of IS D/C met the criteria for DM at year 1 (80% *vs* 84%, *P = *0.77). Interestingly, among patients who were still on IS at 5 years post-diagnosis (*n* = 86), meeting the criteria for DM at 5 years was associated with 2.9 greater odds of achieving D/C at any time beyond that point (OR: 2.93, *P = *0.04). The reasons for not attempting IS D/C (*n* = 36) are described in Supplementary Data S4b, available at *Rheumatology* online.

### Time to immunosuppression discontinuation

In univariate analysis, older age (β = −1.16, *P < *0.001) and membranous LN (β = −22.2, *P = *0.012) were significantly associated with a shorter time to IS D/C, while higher baseline eGFR (β = 0.25, *P = *0.043) and low C3 levels (β = 17.1, *P = *0.032) were significantly associated with longer time.

In multivariate model 1, membranous LN (compared with proliferative LN) was associated with an ∼20-month shorter interval between renal response and IS D/C (β = −19.8, *P = *0.014). We observed a trend for a shorter time to IS D/C for cases treated with MPA (compared with CYC, β = −16.8, *P = *0.051); however, this was not used in the multivariate analysis because of strong collinearity with the LN class (Supplementary Data S3, available at *Rheumatology* online). Using MPA instead of LN class, no association was found with time to IS D/C.

A second multivariate model (model 2) was implemented only for proliferative cases. Age, eGFR, activity index and induction treatment with MPA were included in the model, as they were found to be significantly associated with the time to IS D/C in univariate analysis (data not shown). After adjustments, older age (β = −1.06, *P = *0.009), higher activity index (β = −3.46, *P = *0.002), and induction treatment with MPA (β = −22.1, *P = *0.032) retained their strong association with a shorter time to IS D/C post-renal response. The models are presented in detail in Table 3.

**Table 3.** Baseline determinants (time to discontinuation of immunosuppression after renal response)

	β (95% CI), *P*-value		
Category	Univariate models	Multivariate model 1	Multivariate model 2
Age (per year increase)	**−1.16 (−1.74, −0.57), <0.001**	−0.52 (−1.12, 0.07), 0.08	**−1.06 (−1.84, −0.28), 0.009**
Females	−4.18 (−23.5, 15.1), 0.66		
eGFR (per ml/min/1.73 m^2^ increase)	**0.25 (0.008, 0.50), 0.043**	0.03 (−0.18, 0.25), 0.75	0.11 (**−**0.16, 0.38), 0.42
Proteinuria (per g/24 h increase)	−1.39 (−3.47, 0.69), 0.18		
Membranous class (*vs* proliferative)	**−22.2 (−39.5, −4.99), 0.012**	**−19.8 (−35.6, −4.18), 0.014**	
SLEDAI-2K (per unit increase)	1.53 (−0.26, 3.32), 0.09		
Low C3	**17.1 (1.51, 32.6), 0.032**	11.1 (−3.6, 25), 0.13	
Low C4	0.13 (−16.2, 16.5), 0.98		
Activity Index (per unit increase)^a^	**−2.48 (−4.53, −0.44), 0.018**		**−3.46 (−5.5, −1.38), 0.002**
Chronicity Index (per unit increase)^a^	−3.4 (−9.99, 3.19), 0.30		
Interstitial fibrosis/tubular atrophy (moderate–severe)^b^	−12.2 (−44.1, 19.7), 0.44		
Number of crescents	−0.28 (−2.5, 1.93), 0.80		
Glomerulosclerosis (%)^c^	−0.25 (−0.81, 0.30), 0.37		
Induction treatment (mycophenolic acid)	−16.8 (−33.8, 0.05), 0.051		**−22.1 (−42, −2.1), 0.032**
Time to first CR (months)	−0.39 (−1.03, 0.24), 0.21		

Statistical significance (*P < *0.05) is shown in bold.

a For proliferative LN.

b Refers to the percentage of the renal cortex involved by interstitial fibrosis and tubular atrophy.

c Percentage of sclerosed glomeruli among the total number of glomeruli. β: β-coefficient; CR: complete remission; eGFR: estimated glomerular filtration rate using the CKD-EPI formula; SLEDAI-2K: systemic lupus erythematosus disease activity index.

### Flares after immunosuppression discontinuation

Post-IS D/C flares were observed in 18/75 (18%) patients: 14/18 had renal flares, 4/18 had extrarenal flares (arthritis) and one patient had both renal and extrarenal flares. Remission was re-achieved following treatment in all patients.

While HCQ use at the IS D/C did not appear to protect against post-D/C renal flares, persistent HCQ use (≥2/3 of the time from LN diagnosis until IS D/C) was more frequent in patients without renal flares (55% *vs* 28%, *P = *0.047) (Table 4). Patients with post-D/C renal flares had a higher SLEDAI-2K scores 12 months before IS D/C (median 2 *vs* 0, *P = *0.026) than those without; however, both groups had comparable SLEDAI-2K scores at IS D/C (median 1 *vs* 0, *P = *0.32). No significant differences were observed in baseline serological/immunological tests. In multivariate analysis, including the SLEDAI-2K score at 12 months before IS D/C and persistent HCQ use, the former remained significant (OR: 1.70, *P = *0.013), with a trend for the latter (OR: 0.28, *P = *0.08).

**Table 4.** Differences in characteristics between patients with renal flares after discontinuation of immunosuppressants and those without

Characteristics	Total (*n* = 75)	Without flare (*n* = 61)	With flare (*n* = 14)	*P*-value	Multivariate analysis, OR (95% CI), *P*-value
12 months post diagnosis					
Proteinuria, median (IQR), g/24 h	0.22 (0.34)	0.20 (0.36)	0.30 (2.65)	0.24	
≤0.8, *n* (%)	54 (81.8)	47 (85.5)	7 (63.6)	0.08	
>0.8, *n* (%)	12 (18.2)	8 (14.5)	4 (36.4)		
SLEDAI-2K, median (IQR)	3 (5)	2 (6)	4 (2)	0.73	
Complete remission, *n* (%)	55 (74.5)	45 (75)	10 (71)	0.74	
12 months before immunosuppression discontinuation (IS D/C)					
Proteinuria, median (IQR), g/24 h	0.16 (0.29)	0.12 (0.28)	0.20 (0.28)	0.18	
SLEDAI-2K, median (IQR)	0 (2)	0 (2)	2 (4)	**0.026**	**1.70 (1.12, 2.60), 0.013**
SDI, median (IQR)	0 (0)	0 (0)	0 (0)	0.63	
LLDAS, *n* (%)	46 (92)	34 (92)	12 (92.3)	0.96	
DORIS clinical remission, *n* (%)	46 (90.2)	34 (89.5)	12 (92.3)	0.77	
DORIS complete remission, *n* (%)	26 (52)	22 (57.9)	4 (33.3)	0.13	
At IS D/C					
eGFR, median (IQR), ml/min/1.73 m^2^	108 (28)	110 (41)	94 (18)	0.18	
Proteinuria, median (IQR), g/24 h	0.17 (0.26)	0.16 (0.21)	0.36 (0.55)	0.10	
HCQ use, *n* (%)	44 (59)	38 (62)	6 (42)	0.10	
HCQ persistent use^a^, *n* (%)	37 (49.3)	33 (55)	4 (28)	**0.047**	**0.28 (0.07, 1.10), 0.08**
SLEDAI-2K, median (IQR)	0 (2)	0 (2)	1 (4)	0.32	
SDI, median (IQR)	0 (0)	0 (0)	0 (0)	0.46	
LLDAS, *n* (%)	48 (96)	35 (94.6)	13 (100)	0.39	
DORIS clinical remission, *n* (%)	45 (79)	38 (82.5)	7 (63.6)	0.16	
DORIS complete remission, *n* (%)	45 (79)	38 (82.5)	7 (63.6)	0.17	
Time to first CR, median (IQR), months	7.5 (8.5)	7.5 (8)	7 (15)	0.85	
Sustained CR, median (IQR), months	55 (42)	56 (42)	51 (30)	0.41	
Renal Response to tapering initiation, median (IQR), months	57 (43)	57 (47)	59.5 (30)	0.84	
LN diagnosis to tapering initiation, median (IQR), months	69 (44)	63.5 (50.5)	71.5 (23)	0.64	
Renal flare during tapering, *n* (%)	7 (9.3)	5 (8.2)	2 (14.3)	0.48	
Extrarenal flare during tapering, *n* (%)	2 (2.7)	2 (3.3)	0 (0)	0.99	

Statistical significance (*P < *0.05) is shown in bold.

a Persistent use: ≥2/3 of the follow-up period from LN diagnosis to D/C. CR: complete response; DORIS: Definition of Remission In Systemic Lupus Erythematosus; eGFR: estimated glomerular filtration rate using the CKD-EPI formula; HCQ: hydroxychloroquine; IQR: interquartile range; LLDAS: Lupus Low Disease Activity State; LN: lupus nephritis; OR: odds ratio; SDI: SLICC/ACR Damage Index Score; SLEDAI-2K: systemic lupus erythematosus disease activity index.

In the group of proliferative LN, patients with post-D/C renal flares (*vs* those without) had higher SLEDAI-2K scores 12 months before IS D/C (median 2 *vs* 0; *P = *0.05), lower percentages of persistent HCQ use (33% *vs* 50%, *P = *0.20), and met less frequently the DORIS complete remission criteria (28.6% *vs* 63%, *P = *0.09), although statistically non-significant.

We recorded all renal flares from the initiation of IS tapering onwards, irrespective of IS D/C achievement. In total, 24/111 (21.6%) patients experienced at least one renal flare. Patients with post-IS tapering renal flares received more frequently induction therapy with CYC (81% *vs* 59%, *P = *0.07) and had higher proteinuria levels (≥0.8 g/24 h) at 12 months post-diagnosis (38.1% *vs* 15.8%, *P = *0.02). Patients without flares had higher eGFR levels (109.5 *vs* 94 ml/min/1.73 m^2^, *P = *0.06), met the criteria for DM (85% *vs* 68%, *P = *0.07) and achieved CR (78% *vs* 58.5%, *P = *0.054) more frequently at 12 months post-diagnosis. HCQ use, either at IS tapering initiation or as persistent use (≥2/3 of the time between LN diagnosis and IS tapering initiation), was more common in patients without renal flares (65.8% *vs* 43%, *P = *0.047 and 57.6 *vs* 29.1%, *P = *0.01, respectively) (Table 5). In multivariate analysis, lower proteinuria levels at 12 months post diagnosis (≤0.8 g/24 h, OR: 0.36, *P = *0.07) and persistent HCQ use (OR: 0.40, *P = *0.07) were marginally associated with lower risk of post-IS tapering renal flares.

**Table 5.** Differences between patients who experienced renal flares after any attempt of IS tapering and those who did not

Characteristics	Total (*n* = 111)	Without flare (*n* = 87)	With flare (*n* = 24)	*P*-value	Multivariate analysis, OR (95% CI), *P*-value
At baseline					
Induction treatment (cyclophosphamide), *n* (%)	66 (64)	48 (59)	18 (81)	0.07	
12 months post diagnosis					
Proteinuria, median (IQR), g/24 h	0.26 (0.5)	0.2 (0.37)	0.3 (2.54)	**0.013**	
>0.8, *n* (%)	20 (20.6)	12 (15.8)	8 (38.1)	**0.02**	Reference group
≤0.8, *n* (%)	77 (79.4)	64 (84.2)	13 (61.9)		0.36 (0.12, 1.11), 0.07
eGFR, median (IQR), ml/min/1.73 m^2^	109 (30)	109.5 (24)	94 (41)	0.06	
Complete remission, *n* (%)	81 (73.6)	67 (78)	14 (58.5)	0.054	
Disease modification, *n* (%)	77 (81)	62 (85)	15 (68)	0.07	
At tapering initiation					
HCQ use, *n* (%)	57 (60.5)	48 (65.8)	9 (43)	0.058	
HCQ persistent use^a^, *n* (%)	56 (51)	49 (57.6)	7 (29.1)	**0.01**	0.40 (0.34, 1.11), 0.07

Statistical significance (*P < *0.05) is shown in bold.

a Persistent use: ≥2/3 of the follow-up period from LN diagnosis to tapering initiation. eGFR: estimated glomerular filtration rate using the CKD-EPI formula; HCQ: hydroxychloroquine; IQR: interquartile range.

In patients with proliferative LN, similar findings to those of the entire cohort were observed: patients without flares more frequently maintained persistent HCQ use and were more likely to achieve CR and meet the DM criteria at 12 months post-diagnosis.

### Long-term outcomes

At a median follow-up time of 124 months, only 61.5% (16/26) of patients without IS tapering achieved CR *vs* 81.1% (90/111) of those in whom IS was tapered (*P = *0.03).

**Figure 1.**
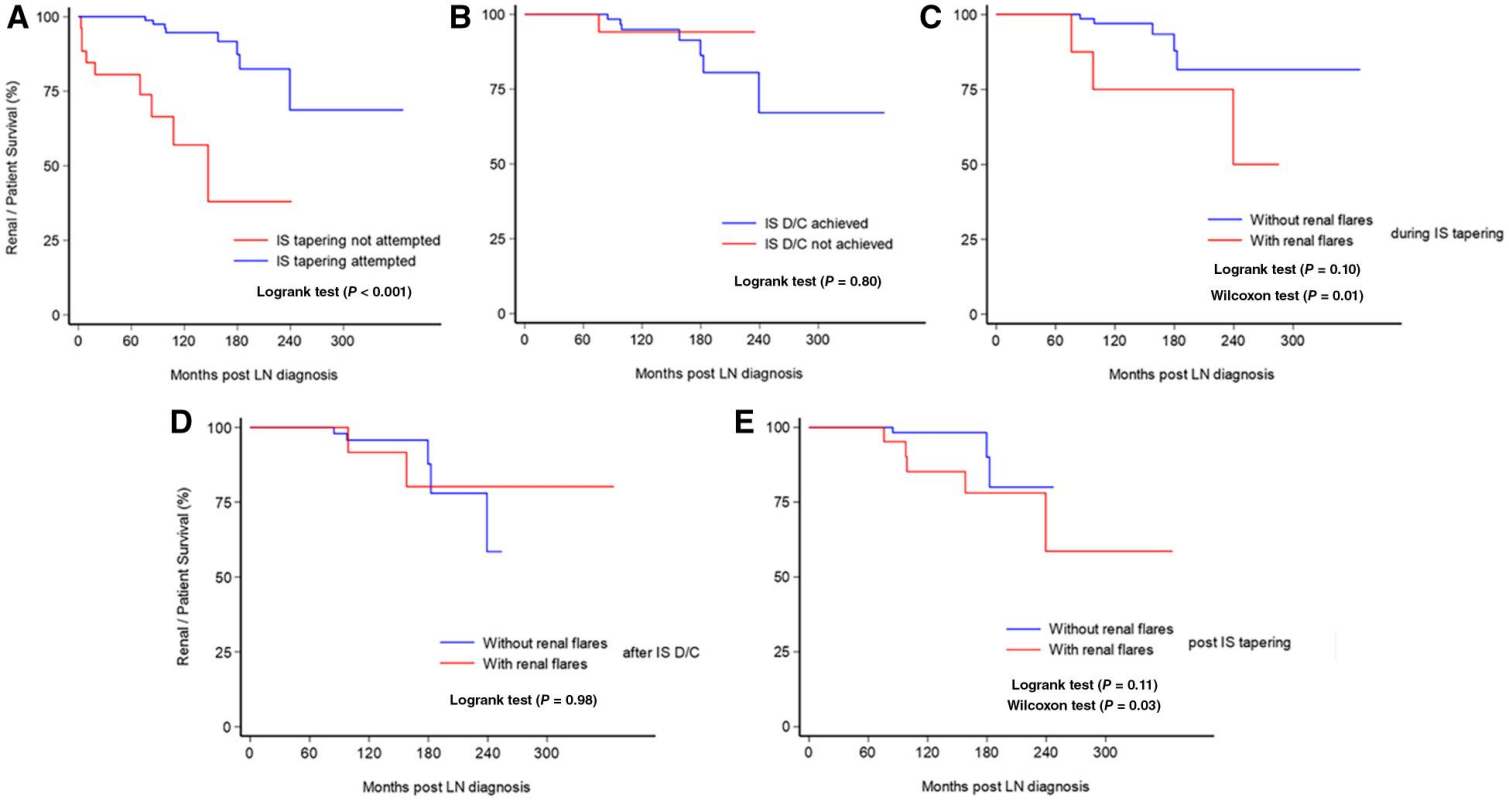
Kaplan–Meier estimates for renal and patient survival according to (**A**) attempt for IS tapering, (**B**) IS D/C achievement, (**C**) renal flare experience during tapering, (**D**) renal flare experience post-IS D/C, and (**E**) renal flare experience at any time after IS tapering initiation. D/C: discontinuation; IS: immunosuppressants; LN: lupus nephritis

Additionally, the probability of ESRD or death (combined due to small number of events) in Kaplan–Meier survival curve analysis (Fig. 1A, *P* < 0.001) and the prevalence of the composite unfavourable outcome [severe eGFR decline compared with baseline (≥30%), chronic kidney disease (CKD), ESRD, and death; Supplementary Data S3, available at *Rheumatology* online; 19.8% *vs* 34.6%, *P = *0.07] were lower among patients in whom IS tapering was attempted. In the EFU, 35% of these patients had SLICC/ACR Damage Index Score (SDI) ≥1 *vs* 52.2% of patients in whom IS tapering was not attempted (*P = *0.09) (Supplementary Table S4, available at *Rheumatology* online).

Although the probability of ESRD or death (Fig. 1B**)** and the prevalence of unfavourable composite outcome (Supplementary Table S4, available at *Rheumatology* online) in IS D/C achievers were comparable to those in non-achievers (*P = *0.80 and *P = *0.34, respectively), the latter group had a lower percentage of CR achievement (72% *vs* 85.5%, *P = *0.09) at the EFU.

Notably, patients who experienced renal flares during IS tapering (*vs* those without) were less likely to be in CR (54.5% *vs* 84%, *P = *0.03), had higher probability of ESRD or death in Kaplan–Meier survival analysis (Fig. 1C) and higher prevalence of the composite unfavourable outcome at the EFU (53% *vs* 16%, *P = *0.008) (Supplementary Table S4, available at *Rheumatology* online).

Patients with renal flares after IS D/C had higher 24-h proteinuria levels (0.35 g/24 h *vs* 0.15 g/24 h, *P = *0.04) and lower rates of CR (64.2% *vs* 90.1%, *P = *0.02) at the EFU, than those without renal flares. However, post-IS D/C flares were not associated with overall ESRD or death rates or the composite unfavourable outcome at the EFU (Fig. 1D, Supplementary Table S4, available at *Rheumatology* online).

A higher prevalence of the unfavourable composite outcome (33.3% *vs* 16%, *P = *0.05) and lower rates of CR at EFU (58.3% *vs* 87.5%, *P = *0.001) were observed when renal flares were examined together during tapering and post-IS D/C. In proliferative LN, CR rates at EFU were similar between patients with and without post-D/C renal flares (78% *vs* 89%, *P = *0.33).

## Discussion

In the current study, IS tapering was attempted in 81% of patients with LN, while IS D/C was achieved in 67.5% of patients at a median time of 57 months after renal response. Flares during the tapering period were the main reason for IS D/C failure, whereas a longer time to first CR and higher SLEDAI-2K score at tapering initiation emerged as predictors of renal flares during tapering. Persistent HCQ use and lower SLEDAI-2K scores 12 months before IS D/C were protective against renal flares after IS D/C. Membranous LN was significantly associated with a shorter time to D/C and a minimal risk of flares. Finally, the occurrence of renal flares during IS tapering predicted unfavourable long-term outcomes.

Treatment of LN aims at remission, prevention of flares and long-term preservation of renal function, while minimizing treatment-related adverse events. However, there is a paucity of data regarding the optimal timing of IS tapering initiation and D/C as well as factors predicting treatment withdrawal achievement.

Data from the Toronto Lupus cohort showed that sustained CR for ≥5 years was associated with reduced rates of flares, CKD, ESRD and death [33]. Moroni *et al.* reported that patients with (*vs* those without) flares after IS D/C had a shorter CR period (12 *vs* 53 months; *P = *0.000) before IS withdrawal [12]. In a study by Zen *et al.*, patients treated with IS for at least 3 years after CR had the lowest risk of flare post-IS D/C (OR = 0.284, *P = *0.023)[16]. Laskari *et al.* have described that tapering of IS <18 months after CR is associated with a 6.3-fold higher risk of flares [34].

To complement the results of previous studies, we investigated possible predictors of flares not only after IS D/C but also during the tapering period. In our cohort, IS tapering was initiated at a median time of ∼3 years after renal response, and renal flares during the tapering period were associated with a longer time to CR post-diagnosis (and not with the duration of CR before tapering initiation) and higher SLEDAI-2K scores at tapering initiation.

Treat-to-target strategies for SLE include LLDAS and DORIS remission. Achieving DORIS remission for at least 24 months demonstrates high specificity (>80%) for reduced damage accrual [35]. In a recent study of 3000 patients with SLE (46.8% with LN), the achievement of DORIS complete remission at the onset of IS tapering was associated with reduced flare rates and prolonged time to flare [36]. In our study, patients achieving *vs* not achieving DORIS complete remission at tapering initiation experienced renal or extrarenal flares less frequently. We also showed that lower SLEDAI-2K scores and proteinuria ≤0.8 g/24 h at month 12 protected against any flares (renal and/or extrarenal) during IS tapering, complementing the results of a previous study from our group [37].

As emphasized in the recent EULAR recommendations for SLE management, tapering and D/C of GCs should precede IS tapering [20]. Our study reinforces this recommendation, demonstrating that patients who remain on GCs at the initiation of IS tapering are at an increased risk of flares. This suggests that even at low doses, GCs may mask ongoing quiescent disease activity. The benefits of HCQ in terms of renal and extrarenal flare prevention are well established [12, 16, 36]. Our study further underscores the critical role of persistent HCQ use in minimizing the risk of disease flares both during the tapering period and post-IS D/C.

Maintenance treatment in patients with pure membranous LN is still debated, especially for those with nephrotic syndrome, but most studies lean towards favouring maintenance, possibly for shorter duration [21]. Of note, in our cohort, patients with membranous LN had a 20-month shorter interval between renal response and IS D/C than patients with proliferative classes. Additionally, the membranous class was associated with minimal post-IS D/C flares and better long-term outcomes, as previously reported by Zen *et al.* [16] and our group [38]. These findings further support the notion that membranous LN may require less exposure to IS treatment than proliferative LN. For patients with proliferative LN, older age, higher activity index, and induction treatment with MPA were strongly associated with a shorter time to IS D/C post-renal response. More specifically, MPA was associated with a >20 months shorter time to IS D/C without an increase in flare risk or adverse long-term outcomes, a finding that is in accordance with our previous reports [38].

Regarding histological lesions, it would be interesting to examine thrombotic microangiopathy/antiphospholipid syndrome nephropathy lesions and their possible correlation with renal outcomes. However, this would require a comprehensive re-evaluation of all renal biopsies, aligning with recent international efforts to better characterize APS nephropathy lesions [39].

Similar to other chronic diseases, DM is an emerging goal in lupus management [31]. In a recent study, DM achievers experienced better long-term renal outcomes than non-achievers [32]. In the present study, we found a correlation between DM achievement and successful IS D/C, supporting the importance of DM in LN management.

We also observed worse long-term outcomes (renal function decline >30% from baseline, CKD, ESRD and/or death) and greater damage accrual by the end of follow-up in patients in whom IS tapering was never attempted. Interestingly, while renal flares during tapering were correlated with adverse long-term outcomes, the post-IS D/C flares were not, in accordance with previous observations of comparable renal SDI damage scores, irrespective of post-IS D/C flare occurrence [16]. However, the limited statistical power due to the small number of events may obscure the association between post-IS D/C flares and long-term outcomes.

The strengths of this study include (i) the use of inception cohort data from two academic centres with a uniform approach; (ii) the investigation of both renal and extrarenal flares, not only after IS D/C, but also during IS tapering; (iii) the assessment of a wide range of potential predictors of the time of IS D/C; and (iv) a long follow-up period of 124 months. This study has some limitations, including its retrospective design and lack of per-protocol biopsies, especially before IS D/C. Such biopsies could offer valuable information about the ideal timing for IS D/C, considering the inconsistencies between clinical and histological remission in LN [40–42]. In our cohort, repeat biopsies were performed based on clinical indications of renal flares or inadequate response to treatment. Moreover, the inclusion of almost exclusively White Europeans and the management of patients in the context of a supportive public health system that provides free medical care for all patients may limit its generalizability to other populations. Finally, although multivariate analyses were conducted, the limited number of flares during tapering or post-IS DC did not enable the inclusion of more than two independent variables in the corresponding models.

In conclusion, our findings underscore the fundamental role of early CR achievement, persistent HCQ use, and the maintenance of optimal low disease activity during follow-up in IS tapering and D/C strategies for LN. Altogether, they can assist in flare-free IS withdrawal, thereby minimizing the risk of adverse long-term outcomes. Lastly, following our results, earlier treatment withdrawal in membranous LN is safe, mitigating the risks associated with exposure to immunosuppressants.

## Supplementary Material

keae381_Supplementary_Data

## Data Availability

Data supporting this study’s findings are available from the corresponding author upon reasonable request.
