# Supplementary material for: Immunosuppressives discontinuation after renal response in lupus nephritis: predictors of flares, time to withdrawal and long-term outcomes
Source: Rheumatology (Oxford). 2024 Jul 22;64(4):1894–903. doi: 10.1093/rheumatology/keae381 (PMC11962912; doi:10.1093/rheumatology/keae381)
Supplement: keae381_Supplementary_Data [file keae381_supplementary_data.zip › keae381_Supplementary_Data/rhe-24-0811-File004.docx]

**Supplementary Material**

**Supplementary Data S1**

*Measured variables*

The following data were collected for each participant: patient and disease characteristics (age, sex, ethnicity, systemic lupus erythematosus [SLE] duration before lupus nephritis [LN], disease activity (measured by the SLE Disease Activity Index 2000– SLEDAI-2K [1], and the Physician Global Assessment – PGA [2]), disease chronicity (measured by SLICC/ACR Damage Index Score – SDI [3]), renal flares, extra-renal flares, laboratory results [total blood count, C3, C4, anti-ds-DNA, serum creatinine (SCreat), estimated glomerular filtration rate (eGFR), 24-hour (24-h) proteinuria, urine sediment], histological characteristics (LN class, activity index, chronicity index, crescents, interstitial fibrosis/tubular atrophy [IF/TA], glomerulosclerosis), and treatment regimens. The selection, dosage, and duration of each treatment were determined at the physician’s discretion. Finally, the attainment of disease modification was recorded for each patient at 12, 60- and 72-months post-diagnosis [4, 5].

**Supplementary Data S2**

*Definitions*

Renal response and flares were defined in accordance with the 2012 EULAR/ERA-EDTA guidelines [6] and the 2012 KDIGO recommendations [7]: complete response (CR) as proteinuria below 500 mg/24-h and serum creatinine within 10% of the baseline. Partial Response (PR) was defined as a ≥50% reduction in proteinuria below the nephrotic range and a serum creatinine level within 10% of the baseline. Renal flares were defined as ≥10 red blood cells per high-power field (RBCs/hpf) increase in glomerular hematuria, with or without a ≥10% decline in eGFR (classified as nephritic flares). A proteinuric flare was defined as the reproducible doubling of proteinuria to >1000 mg/24-h if CR had been achieved previously or to ≥2000 mg/24-h if PR had been achieved. Stage III-IV chronic kidney disease (CKD) was characterized by an eGFR between 15-60 mL/min/1.73 m², while end stage kidney disease (ESRD) by an eGFR <15 mL/min/1.73 m² or by the initiation of renal replacement therapy. SLE remission targets were evaluated using the Lupus Low Disease Activity State (LLDAS, characterized by SLEDAI-2K ≤4, PGA ≤1, and prednisolone ≤7.5 mg/day) and the definition of remission in SLE (DORIS clinical remission, defined by clinical SLEDAI-2K=0, PGA score <0.5, and prednisolone dose ≤5 mg/day; DORIS complete remission on therapy as SLEDAI-2K=0, PGA score <0.5, and prednisolone ≤5 mg/day) [8, 9].

**Supplementary Data S3**

*Statistical Analysis*

Selection of independent variables on multivariate models and multicollinearity issues, modelling for time to immunosuppression (IS) discontinuation (D/C) after renal response, definition of the composite unfavorable outcome at the end of follow up (EFU).

For the analysis on the time to IS D/C, only the characteristics at the time of LN diagnosis and time to first CR were included in the model (as independent variables), since longer timepoints could not serve as predictors of that time.

Prior to performing any multivariate model on the flares during IS tapering (due to small number of events), multicollinearity issues were addressed among the statistical significant variables of the univariate models, using appropriate tests including chi-square, Cramer’s V coefficient (V), Spearman correlation coefficient (r_s_), and Mann–Whitney U test, (**Supplementary Table S1**). Two multivariate models were conducted using SLEDAI-2K score at tapering initiation in both models (independent of any other variable), with time to first CR (Model 1) and renal flares before tapering (Model 2) for the analysis of predictors of flares during tapering. Model 1 was selected, because of lower value of the Akaine Information Criterion (AIC) (38 vs. 52 in model 2), indicating a better model fit.

In the univariate analysis for time to IS D/C, induction treatment showed a trend towards significance (MPA vs. CYC: β-coef=-16.8, p=0.051). Due to strong collinearity with LN class (Chi-square test, p=0.001; Cramer’s V test, p=0.28), induction treatment was not included in the multivariate analysis.

Two multivariate linear models were conducted for the analysis on the time to ISD/C after renal response. The first linear model (Model A) in all 75 patients who discontinued IS therapy and the second (Model 2) among the 54 patients with proliferative LN who discontinued IS therapy. Low C3 was not statistically significant in the univariate analysis among proliferative LN; in contrast, treatment with mycophenolic acid was significant in the univariate analysis in patients with proliferative LN.

In the analysis on the EFU, we generated the Kaplan Meier survival curves for the composite outcome of ESRD and death as these represent the primary hard outcomes in LN, and we defined the composite unfavorable outcome of severe eGFR decline compared to baseline (≥30%), progression to stage IV CKD, ESRD, and death, as adverse long-term outcomes, due to the small number of the aforementioned events in the cohort.

**Supplementary Data S4**

1. The reasons for not attempting IS tapering (n=26) were the following: absence of renal response (n=9), multiple renal flares during follow-up (n=3), extrarenal disease activity (central nervous system involvement, gastrointestinal vasculitis, arthritis, and rash) (n=5), non-compliance (n=4), or physician’s judgment for not tapering (n=9) due to disease duration of less than three years). Four patients had ≥1 reasons for not initiating tapering. No specific histological class was associated with the decision not to attempt IS tapering.
2. Among the 36 patients who didn’t discontinue IS treatment, 18 were still on tapering, 9 were in remission (following a renal flare during the tapering period), but without re-initiating IS tapering per clinician’s decision, 13 were still on IS tapering due to prior extrarenal disease activity (CNS involvement in 3/13, gastrointestinal vasculitis in 1/13, liver involvement in 1/13, pancreas involvement in 1/13, serositis in 1/13, arthritis with rash in 6/13 cases), and 2 were noncompliant. Six patients had ≥1 reasons for not discontinuing the IS treatment.

**Supplementary Data S5**

*Outcomes on extrarenal flares*

*Flares during immunosuppression (IS) tapering*

Patients with extrarenal flares during IS tapering (compared to those without) were more likely to receive GCs (87.5% vs. 50%, p=0.048) at tapering initiation (median dose of 2 mg/24-h) and experience extrarenal flares before tapering initiation (33.3% vs. 4%, p=0.02). No other differences were observed between the two groups regarding their characteristics at baseline, month 12, or time of tapering initiation (data not shown).

When renal and extrarenal flares were investigated as a composite outcome, patients without flares during tapering had lower 24-h proteinuria (median 0.20 vs. 0.48 g/24-h, p=0.03) and lower SLEDAI-2K scores (median 2 vs. 4, p=0.05) at 12 months post-diagnosis, compared to those with flares. Patients who experienced flares during tapering (vs. those who did not) were more likely to have higher SLEDAI-2K scores (median 2 vs. 0, p=0.01), not achieve DORIS complete remission (26.7% vs. 53.8%, p=0.05), and receive GCs (87.6% vs. 48.1%, p=0.009) at the time of tapering initiation (**Supplementary Table 2**). No other differences were observed at any of the assessed time points.

Patients with proliferative LN experiencing extrarenal flares during IS tapering (compared to those without) were more likely to receive GCs (100% vs. 50.8%, *p-value*=0.033) at tapering initiation. Previous experience of extrarenal flares, before tapering initiation, did not retain its significance (*p-value*=0.81).

*Flares after immunosuppression discontinuation (IS D/C)*

Post-IS D/C extrarenal flares occurred more frequently in patients who experienced extrarenal flares both before and during the IS tapering period (20% vs. 1.4%, p=0.01).

In the subgroup analysis of patients with proliferative LN, no significant differences were observed in the incidence of extrarenal flares post-D/C.

A similar analysis for all extrarenal flares from the initiation of IS tapering onwards, irrespective of IS D/C achievement or not, was developed. In total, 12/111 (10.8%) patients experienced at least one extrarenal flare following any dose reduction in IS. All patients who experienced a flare were females (100 vs 77.8% of those without flares, p-value=0.06), and had low C3 at baseline (100 vs 70.2%, p-value=0.056). No other difference was observed at any time point of the follow-up.

*All flares (renal, extrarenal) post any IS tapering initiation (during tapering and after D/C)*

Overall, 33 patients (29.7%) experienced at least one flare, renal or extrarenal, following any IS tapering attempt. Patients who experienced either type of flare were more likely to have low C3 or C4 levels at baseline (82% vs. 69%, p=0.10 and 85.7% vs. 63%, p=0.03, respectively) and higher proteinuria (32% vs. 15% with >0.8 g/24h, p=0.06) 12 months post-diagnosis. At the time of tapering initiation, these patients were more frequently on GCs (75% vs. 45%, p=0.01) and had higher SLEDAI-2K scores (2 vs. 0, p=0.08). While HCQ use at the time of IS D/C did not appear to protect against post-tapering flares, persistent HCQ use (≥2/3 of the time from LN diagnosis until IS tapering initiation) was more common in patients without flares (57% vs. 37%, p=0.06). No significant differences were observed in the incidence of any type of flare (renal, extrarenal, or combined) between patients with proliferative or membranous LN.

*Long-term outcomes*

Extrarenal flares were not associated with unfavorable long-term outcomes either during tapering or after IS D/C.

**Supplementary Table S1.** Tests examining correlation between significant variables in the analysis of renal flares during IS tapering.

|  | SLEDAI-2K (12 months post-diagnosis) | CR (12 months post-diagnosis) | DM (12 months post-diagnosis) | SLEDAI-2K (at tapering initiation) | Time to first CR (months) | Renal flare before tapering |
| --- | --- | --- | --- | --- | --- | --- |
| Proteinuria (12 months post-diagnosis, g/24h) | Spearman Correlation Coefficient r_s_=0.46 | Mann−Whitney U (p<0.001) | Mann−Whitney U (p=0.001) | Spearman Correlation Coefficient  r_s_=-0.12 | Spearman Correlation Coefficient r_s_=0.55 | Mann−Whitney U (p=0.007) |
| SLEDAI-2K (12 months post-diagnosis) | - | Mann−Whitney U (p<0.001) | Mann−Whitney U (p=0.005) | Spearman Correlation Coefficient r_s_=0.14 | Spearman Correlation Coefficient r_s_=0.56 | Mann−Whitney U (p=0.0006) |
| CR (12 months post-diagnosis) | - | - | Chi-square (p=0.01) Cramer’s V=-0.26 | Mann−Whitney U (p=0.67) | Mann−Whitney U (p<0.001) | Chi-square (p=0.01) Cramer’s V=0.25 |
| DM (12 months post-diagnosis) | - | - | - | Mann−Whitney U (p=0.10) | Mann−Whitney U (p=0.16) | Chi-square (p=0.53) Cramer’s V=-0.06 |
| SLEDAI-2K (at tapering initiation) | - | - | - | - | Spearman Correlation Coefficient  r_s_=-0.14 | Mann−Whitney U (p=0.39) |
| Time to first CR (months) | - | - | - | - | - | Mann−Whitney U (p=0.27) |

CR: complete remission, DM: disease modification, SLEDAI-2K: systemic lupus erythematosus disease activity index

**Supplementary Table S2.** Differences between patients who experienced flares (renal/extrarenal) during tapering and those who did not.

| **Characteristics** | **Total  (n=111)** | **Without Flare (n=92)** | **With Flare (n=19)** | **p-value** |
| --- | --- | --- | --- | --- |
|  | **Median [IQR], N (%)** | | |  |
| ***12 months post diagnosis*** |  |  |  |  |
| Proteinuria (g/24-h) | 0.26 [0.50] | 0.20 [0.34] | 0.48 [1.65] | **0.03** |
| - ≤0.8 (g/24-h) | 77 (79.4) | 67 (82.7) | 10 (62.5) | 0.05 |
| - >0.8 (g/24-h) | 20 (20.6) | 14 (17.3) | 6 (37.5) |  |
| SLEDAI-2K | 4 [5] | 2 [4] | 4 [4] | 0.05 |
| Complete Remission | 81 (73.6) | 70 (76.9) | 11 (57.9) | 0.08 |
| ***At tapering initiation*** |  |  |  |  |
| GC use | 51 (54.3) | 38 (48.1) | 13 (86.7) | **0.009** |
| GC (mg/24-h) | 4 [2] | 4 [1] | 4 [2] | 0.13 |
| SLEDAI-2K | 0 [2] | 0 [2] | 2 [2] | **0.01** |
| LLDAS | 90 (96.8) | 76 (97.4) | 14 (93.3) | 0.41 |
| DORIS clinical remission | 89 (95.7) | 75 (96.2) | 14 (93.3) | 0.51 |
| DORIS complete remission | 46 (49.5) | 42 (53.8) | 4 (26.7) | 0.05 |

Statistical significance (p < 0.05) is shown in bold

DORIS: Definition of Remission In Systemic Lupus Erythematosus, GC: glucocorticoids, IQR: interquartile range, N: number, LLDAS: Lupus Low Disease Activity State, SLEDAI-2K: systemic lupus erythematosus disease activity index

**Supplementary Table S3.** Differences between patients with proliferative LN who experienced renal flares during tapering and those who did not.

| **Characteristics** | **Total  (n=78)** | **Without Flare (n=68)** | **With Flare (n=10)** | **p-value** |
| --- | --- | --- | --- | --- |
|  | **Median [IQR], N (%)** | | |  |
| ***At baseline*** |  |  |  |  |
| Induction Treatment (Cyclophosphamide) | 56 (73.7) | 47 (70.1) | 9 (100) | 0.056 |
| ***12 months post diagnosis*** |  |  |  |  |
| Proteinuria (g/24-h) | 0.20 [0.70] | 0.2 [0.36] | 0.50 [2.47] | 0.12 |
| - ≤0.8 | 52 (77.6) | 47 (81) | 5 (55.5) | 0.07 |
| - >0.8 | 15 (22.4) | 11 (19) | 4 (44.5) |  |
| SLEDAI-2K | 4 [4] | 4 [4] | 4 [6] | 0.17 |
| Complete Remission | 55 (71.4) | 51 (76) | 4 (40) | **0.02** |
| Disease Modification | 54 (81.8) | 50 (87.7) | 4 (44.4) | **0.002** |
| ***At tapering initiation*** |  |  |  |  |
| HCQ use | 41 (59.4) | 38 (62.3) | 3 (37.5) | 0.12 |
| GC use | 37 (54.4) | 31 (50.8) | 6 (85.7) | 0.07 |
| GC (mg/24-h) | 4 [2] | 4 [2] | 4 [2] | 0.17 |
| SLEDAI-2K | 2 [2] | 0 [2] | 2 [2] | 0.11 |
| ***Time to first Complete Remission (months)*** | 6 [13] | 6 [10] | 12 [32] | **0.02** |
| ***Renal Response to tapering initiation (months)*** | 34 [35] | 32 [31] | 54 [18] | 0.08 |
| ***LN diagnosis to tapering initiation (months)*** | 43 [32] | 43 [32] | 60 [55] | 0.07 |
| ***At least one renal flare before tapering*** | 18 (23) | 14 (20) | 4 (40) | 0.17 |

Statistical significance (p < 0.05) is shown in bold

SLEDAI-2K: systemic lupus erythematosus disease activity index, HCQ: hydroxychloroquine, GC: glucocorticoids, LN: lupus nephritis, IQR: interquartile range, N: number

**Supplementary Table S4.** Differences in long-term outcomes in patients with different immunosuppression strategies and flare occurrences during follow-up.

| **Outcome in EFU** | **Composite Unfavorable Outcome ^a^** | **CR** | **SDI≥1** |
| --- | --- | --- | --- |
|  | **N (%), p-value** | | |
| **IS tapering attempted vs. Not attempted** | 22 (19.8) vs. 9 (34.6) 0.07 | **90 (81.1) vs. 16 (61.5) 0.03** | 37 (35) vs. 12 (52.2) 0.09 |
| **IS D/C  vs.  Not D/C** | 13 (17.3) vs. 9 (25) 0.34 | 64 (85.5) vs. 26 (72) 0.09 | 27 (38) vs. 10 (28.6) 0.33 |
| **With Renal Flares  vs.  Without at IS tapering** | **6 (53) vs. 16 (16) 0.008** | **6 (54.5) vs. 84 (84) 0.03** | 5 (50) vs. 32 (33.3) 0.29 |
| **With Renal Flares vs.  Without post-IS D/C** | 3 (21.4) vs. 10 (16.4) 0.70 | **9 (64.2) vs. 55 (90.1) 0.02** | 5 (41.6) vs. 22 (37.3) 0.75 |
| **With Renal Flares vs.  Without post-IS tapering^b^** | 8 (33.3) vs. 14 (16) 0.05 | **14 (58.3) vs. 76 (87.5) 0.001** | 9 (42) vs. 28 (32) 0.39 |

Statistical significance (p<0.05) is shown in bold

CR: complete remission, D/C: discontinuation, EFU: end of follow up, IS: immunosuppression, N: number, SDI: SLICC/ACR Damage Index Score, vs.: versus, N: number

a: Composite unfavorable outcome: severe eGFR decline compared to baseline (≥30%), chronic kidney disease, end stage renal disease, and death

b: all flares post any IS tapering initiation (during tapering and after D/C)

**Supplementary Figure S1.** Study Flowchart


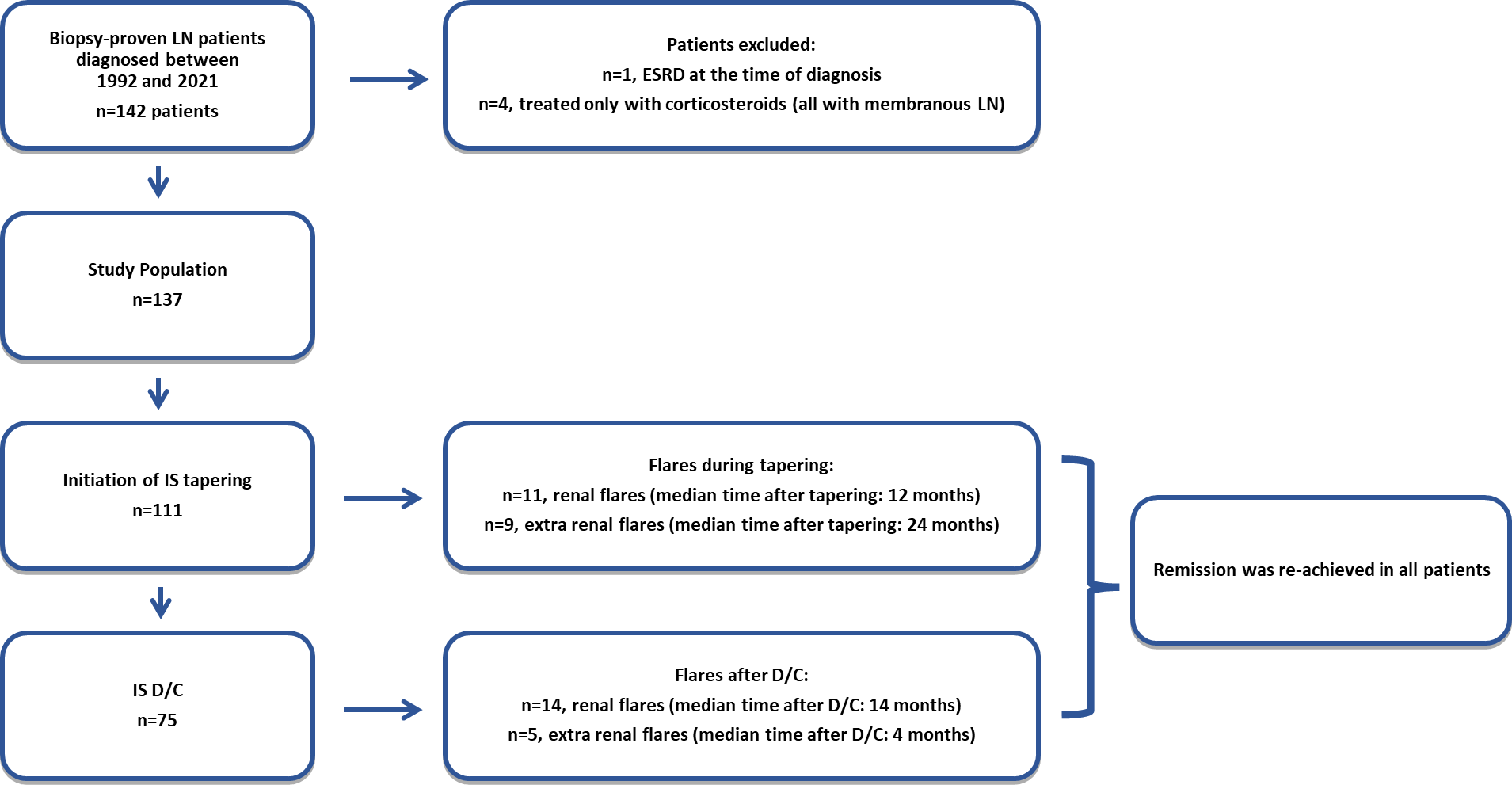


n: number, IS: immunosuppression, D/C: discontinuation, LN: lupus nephritis, ESRD: end stage renal disease

**References**

1. Gladman DD, Ibañez D, Urowitz MB (2002) Systemic lupus erythematosus disease activity index 2000. J. Rheumatol. 29:

2. Petri M, Genovese M, Engle E, Hochberg M (1991) Definition, incidence, and clinical description of flare in systemic lupus erythematosus. A prospective cohort study. Arthritis Rheum 34:937–944

3. Gladman DD, Urowitz MB, Goldsmith CH, et al (1997) The reliability of the Systemic Lupus International Collaborating Clinics/American College of Rheumatology damage index in patients with Systemic Lupus Erythematosus. Arthritis Rheum 40:809–813

4. van Vollenhoven R, Askanase AD, Bomback AS, et al (2022) Conceptual framework for defining disease modification in systemic lupus erythematosus: a call for formal criteria. Lupus Sci Med 9:e000634

5. Panagiotopoulos A, Kapsia E, Michelakis I, Boletis J, Marinaki S, Sfikakis PP, Tektonidou MG (2023) Disease modification achievement in patients with lupus nephritis in a real-life setting: mission impossible? RMD Open 9:e003158

6. Bertsias GK, Tektonidou M, Amoura Z, et al (2012) Joint European League Against Rheumatism and European Renal Association–European Dialysis and Transplant Association (EULAR/ERA-EDTA) recommendations for the management of adult and paediatric lupus nephritis. Ann Rheum Dis 71:1771–1782

7. Cattran DC, Feehally J, Cook HT, et al (2012) Kidney Disease: Improving Global Outcomes (KDIGO) Glomerulonephritis Work Group. KDIGO Clinical Practice Guideline for Glomerulonephritis. Kidney Int Suppl 2:139

8. Franklyn K, Lau CS, Navarra S V., et al (2016) Definition and initial validation of a Lupus Low Disease Activity State (LLDAS). Ann Rheum Dis 75:1615–1621

9. van Vollenhoven RF, Bertsias G, Doria A, et al (2021) 2021 DORIS definition of remission in SLE: final recommendations from an international task force. Lupus Sci Med 8:e000538
