# Supplementary figures and images for: Immunosuppressives discontinuation after renal response in lupus nephritis: predictors of flares, time to withdrawal and long-term outcomes
Source: Rheumatology (Oxford). 2024 Jul 22;64(4):1894–903. doi: 10.1093/rheumatology/keae381 (PMC11962912; doi:10.1093/rheumatology/keae381)

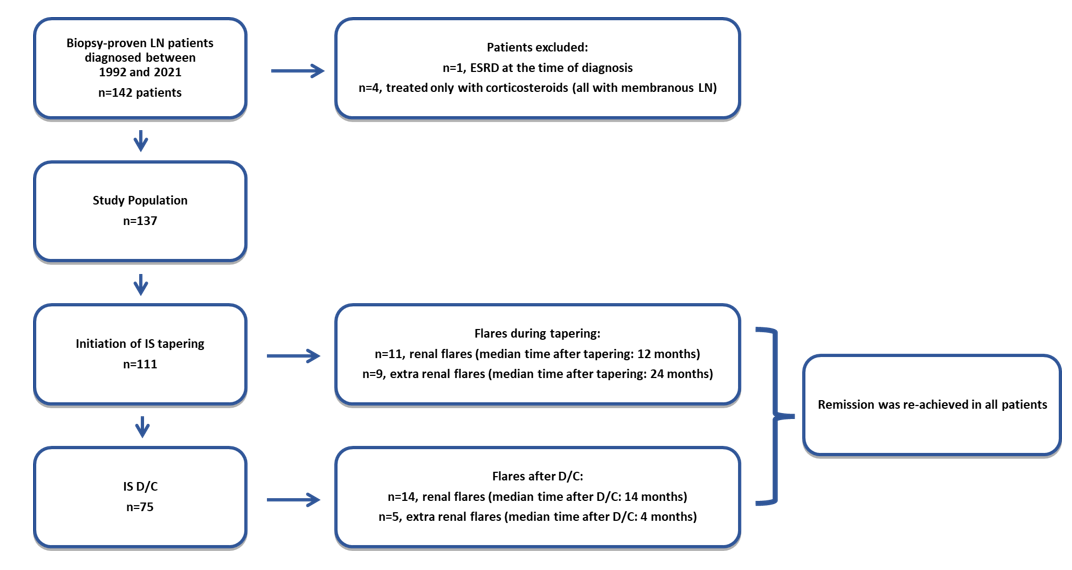

Supplement: keae381_Supplementary_Data [file keae381_supplementary_data.zip › keae381_Supplementary_Data/rhe-24-0811-File005.tiff]
